# The effect of eruption guidance appliances on occlusal traits: a systematic review and meta-analysis

**DOI:** 10.1093/ejo/cjag020

**Published:** 2026-05-11

**Authors:** Lotta Pelkonen, Auli Suominen, Anna-Liisa Svedström-Oristo

**Affiliations:** Institute of Dentistry, University of Turku, Lemminkäisenkatu 2, Turku 20520, Finland; Department of Community Dentistry, Institute of Dentistry, University of Turku, Lemminkäisenkatu 2, Turku 20520, Finland; Department of Pediatric Dentistry and Orthodontics, Institute of Dentistry, University of Turku, Lemminkäisenkatu 2, Turku 20520, Finland

**Keywords:** Angle Class II, mixed dentition, overbite, overjet, prefabricated appliance

## Abstract

**Background:**

Early treatment of malocclusion in primary and early mixed dentition is often carried out by dentists under the supervision of orthodontists. In these treatments, simple appliances are preferred.

**Objectives:**

To analyse which occlusal traits can be corrected with prefabricated eruption guidance appliances (EGAs) in mixed dentitions.

**Search Methods:**

Articles written in English and published between January 1990 and March 2023 were searched from Cochrane Library, Clinicaltrials.gov, EMBASE, International Clinical Trials Registry Platform, LILAC, PubMed, and Scopus.

**Selection Criteria:**

The inclusion criteria were formed according to the Patient, Intervention, Comparison, Outcomes, and Study design schema (PICOS).

**Data Collection and Analysis:**

Selection of the articles followed the Preferred Reporting Items for Systematic Reviews and Meta-Analyses (PRISMA) guidelines. Risk of bias was evaluated using RoB 2 and ROBINS-I tools. The pooled estimates for the change in clinical and cephalometric measurements were calculated. The overall quality of the outcome was assessed using the Grading of Recommendations Assessment, Development, and Evaluation (GRADE) tool.

**Results:**

Seven articles with 277 patients and 219 controls were included. One was a randomized controlled trial, one was a retrospective study, and three were prospective treatment studies. Two follow-up studies analysed cephalometric data of already included studies. Four prefabricated appliances were used: Education Fonctionnelle, LM-Activator™, Nite-Guide^®^, and Occlus-o-Guide^®^. Treatment protocols varied from passive night-time use to combinations of active daytime and passive night-time use. Treatment periods varied from 10 months to 3.3 years. The main findings were reductions in overjet (range 1.1–2.3 mm) and overbite (range 0.6–2.0 mm). Other findings included improvement in crowding and molar relationship and better tooth-to-tooth contacts. Even though most of the changes were dental, significant increases in the length of the mandible were reported in two studies. Four studies were included in the meta-analyses. In these analyses, the changes in overjet, overbite, SNB, and ANB were found statistically significant.

**Conclusions:**

EGAs comprise a group of functional appliances suitable for reduction of excessive overjet and overbite in mixed dentition. However, more studies are needed to elucidate their effects on skeletal structures.

**Registration:**

The protocol for this review was registered at PROSPERO under the ID CRD42023441197.

## Introduction

Malocclusion is common already in primary dentitions, occurring in about 54% of 3–6-year-old children [1]. In Finland, the most common indications for orthodontic treatment in the primary dentition are anterior and lateral crossbites and traumatic deep bite, while in the early mixed dentition, the most common indications include crowding, crossbites, and severe Class II malocclusion [2]. According to previous studies [3, 4], occlusal deviations diagnosed early, like distal step or Angle Class II molar relationship, have only a minor tendency to improve without orthodontic treatment. Early treatment enables the use of growth and guidance of tooth eruption as tools for correction of deviating occlusal traits.

In Finland, eruption guidance appliances (EGAs) became more prevalent at the end of the 1990s, and their use in public health centres became established quickly. In the early 2000s, the EGA was the most used appliance in primary dentition and third in early mixed dentition [2]. According to a recent article, the EGA is currently the second-most common appliance among 5–11-year-old children [5].

The EGA is a prefabricated, activator-type appliance, typically fabricated from medical-grade silicone. Compared to other removable orthodontic appliances, it exhibits greater softness and flexibility. The mandibular part of the appliance has been positioned slightly forward to obtain Angle Class I intercuspation. The EGAs can be used to guide the eruption of permanent teeth into their optimal positions, enabling good contact between upper and lower teeth. Manufacturers provide appliances in different configurations for primary and permanent dentitions, including different sizes and levels of stiffness. The structure of the EGAs and instructions for their use vary according to the brand and goal of treatment.

Over the years, the skeletal and dental effects of EGAs have been presented in case reports [6], and their capability in treatment of Angle Class II division 1 malocclusion has been compared with that of other appliances. These include the Fränkel appliance [7], Twin Block, Activator-Headgear [8], and, more recently, the Hyrax appliance [9]. In the retrospective studies [7, 8], the effects of EGAs were found to be comparable to those of control appliances. In the prospective study [9], changes in the mandibular length and in angles SNA and SNB were significantly bigger in the EGA group.

However, even though there are good clinical experiences of using EGAs, scientific evidence of their effects is limited. Although some recently published systematic reviews and meta-analyses have focused on the effectiveness of early treatment on myofunctional factors and malocclusion [10–12], they include one to four studies with EGAs and analyse different dental stages. Comparisons are made with other appliances [10–12], untreated controls [10, 12], and patients in different treatment phases [12].

Therefore, the focus question in this systematic review and meta-analysis was ‘What kind of occlusal traits can be improved with EGAs’. The primary aim was to analyse dental changes, and the second to evaluate whether there was evidence of skeletal changes too.

## Materials and methods

### Study protocol and registration

The protocol of this review was registered at PROSPERO under the ID CRD42023441197.

### Eligibility criteria

The main question of ‘What kind of occlusal traits can be improved with EGAs’ was formulated according to the Patient, Intervention, Comparison, Outcomes, and Study design model (PICOS) to aid the selection of eligible studies. The inclusion criteria were as follows:

P: Mixed dentition in generally healthy children and adolescents
I: Orthodontic treatment with a prefabricated EGA
C: A control group with similar age and sex distribution without orthodontic treatment history
O: Improvement in occlusal traits (overjet, overbite, crossbite, scissor bite, crowding, spacing, molar relationship, other) and skeletal changes (sagittally and vertically) from the beginning (T1) to the completion of the study (T2)
S: Randomized controlled trials, cohort studies, and non-randomized controlled trials

Only studies published in English were included.

The exclusion criteria included reviews; children and adolescents with cleft lip and/or palate; any other syndromes or medical disorders affecting the craniofacial complex; case reports; studies with no control group; and personalized orthodontic appliances.

### Information sources and search strategy

Searches were made systematically in seven electronic databases: Cochrane Library, Clinicaltrials.gov, EMBASE, International Clinical Trials Registry Platform, LILAC, PubMed, and Scopus. The search period was limited to January 1990–March 2023. In addition, the reference lists of all suitable articles were hand searched. The search terms are listed in Table 1.

**Table 1:** Search terms used in the seven electric databases.

Database	Search format
**Scopus Embase**	TITLE-ABS-KEY ((orthodontics OR ‘ortho* treatment’ OR ‘orthodontic retention’ OR ‘orthodontic follow-up’) AND (‘eruption guidance appliance*’ OR ega OR positioner OR ‘LM activator’ OR ‘EGA therapy’ OR ‘myofunctional appliance’) AND (‘class 1 malocclusio*’ OR ‘class II’ OR malocclusio* OR overjet OR overbite OR crowding OR crossbite OR ‘lateral crossbite’ OR ‘reverse overjet’ OR ‘anterior crossbite’ OR ‘scissor bite’ OR ‘lingual crossbite’ OR ‘molar relationship’ OR alignment OR ‘incisor alignment’) AND (tooth OR teeth OR molar* OR premolar* OR incisor* OR canine*) OR (‘deciduous dentition’ OR ‘primary dentition’ OR ‘early permanent dentition’ OR ‘permanent dentition ‘OR ‘early treatment’ OR ‘late treatment’) OR (child* OR adolescent* OR adult*) AND NOT (‘aligner* therapy’ OR ‘aligner* treatment’))
**PubMed**	(‘Orthodontics’ [Mesh] OR orthodontics OR ‘orthodontic treatment’ OR retention OR ‘Follow-Up Studies’ [Mesh] OR ‘orthodontic follow-up’) AND (‘eruption guidance appliance*’ OR EGA OR positioner OR ‘LM activat*’ OR ‘myofunctional appliance’) AND (‘Malocclusion, Angle Class I’ [Mesh] OR ‘class I’ OR malocclusio* OR ‘Malocclusion, Angle Class II’ [Mesh] OR ‘class II’ OR overjet OR overbite OR crowding OR crossbite OR ‘lateral crossbite’ OR ‘reverse overjet’ OR ‘anterior crossbite’ OR ‘scissor bite’ OR ‘lingual crossbite’ OR ‘molar relationship’ OR alignment OR ‘incisor alignment’) AND (‘Dentition, Mixed’ [Mesh] OR dentition OR tooth OR teeth OR molar* OR premolar* OR incisor* OR canine* OR ‘deciduous dentition’ OR ‘primary dentition’ OR ‘early permanent dentition’ OR ‘permanent dentition’ OR ‘early treatment’ OR ‘late treatment’ OR child* OR adolescent* OR adult*)
**LILAC**	(orthodontics OR ‘ortho* treatment’ OR ‘orthodontic retention’ OR ‘orthodontic follow-up’) AND (‘eruption guidance appliance*’ OR ega OR positioner OR ‘LM activator’ OR ‘EGA therapy’ OR ‘myofunctional appliance’) AND (‘class 1 malocclusio*’ OR ‘class II’ OR malocclusio* OR overjet OR overbite OR crowding OR crossbite OR ‘lateral crossbite’ OR ‘reverse overjet’ OR ‘anterior crossbite’ OR ‘scissor bite’ OR ‘lingual crossbite’ OR ‘molar relationship’ OR alignment OR ‘incisor alignment’) AND (tooth OR teeth OR molar* OR premolar* OR incisor* OR canine*) OR (‘deciduous dentition’ OR ‘primary dentition’ OR ‘early permanent dentition’ OR ‘permanent dentition ‘OR ‘early treatment’ OR ‘late treatment’) OR (child* OR adolescent* OR adult*) AND NOT (‘aligner* therapy’ OR ‘aligner* treatment’)
**ICTRP**	(‘Orthodontics’ OR orthodontics OR ‘orthodontic treatment’ OR retention OR ‘Follow-Up Studies’ OR ‘orthodontic follow-up’) AND (‘eruption guidance appliance*’ OR EGA OR positioner OR ‘LM activat*’ OR ‘myofunctional appliance’) AND (‘Malocclusion, Angle Class I’ OR ‘class I’ OR malocclusio* OR ‘Malocclusion, Angle Class II’ OR ‘class II’ OR overjet OR overbite OR crowding OR crossbite OR ‘lateral crossbite’ OR ‘reverse overjet’ OR ‘anterior crossbite’ OR ‘scissor bite’ OR ‘lingual crossbite’ OR ‘molar relationship’ OR alignment OR ‘incisor alignment’) AND (‘Dentition, Mixed’ OR dentition OR tooth OR teeth OR molar* OR premolar* OR incisor* OR canine* OR ‘deciduous dentition’ OR ‘primary dentition’ OR ‘early permanent dentition’ OR ‘permanent dentition’ OR ‘early treatment’ OR ‘late treatment’ OR child* OR adolescent* OR adult*)
**Cochrane Library**	(‘Orthodontics’ [Mesh] OR orthodontics OR ‘orthodontic treatment’ OR retention OR ‘Follow-Up Studies’ [Mesh] OR ‘orthodontic follow-up’) AND (‘eruption guidance appliance*’ OR EGA OR positioner OR ‘LM activat*’ OR ‘myofunctional appliance’) AND (‘Malocclusion, Angle Class I’ [Mesh] OR ‘class I’ OR malocclusio* OR ‘Malocclusion, Angle Class II’ [Mesh] OR ‘class II’ OR overjet OR overbite OR crowding OR crossbite OR ‘lateral crossbite’ OR ‘reverse overjet’ OR ‘anterior crossbite’ OR ‘scissor bite’ OR ‘lingual crossbite’ OR ‘molar relationship’ OR alignment OR ‘incisor alignment’) AND (‘Dentition, Mixed’ [Mesh] OR dentition OR tooth OR teeth OR molar* OR premolar* OR incisor* OR canine* OR ‘deciduous dentition’ OR ‘primary dentition’ OR ‘early permanent dentition’ OR ‘permanent dentition’ OR ‘early treatment’ OR ‘late treatment’ OR child* OR adolescent* OR adult*)
**Clinicaltrials.gov**	‘malocclusion’ AND ‘myofunctional appliance’

### Study selection

**Figure 1:**
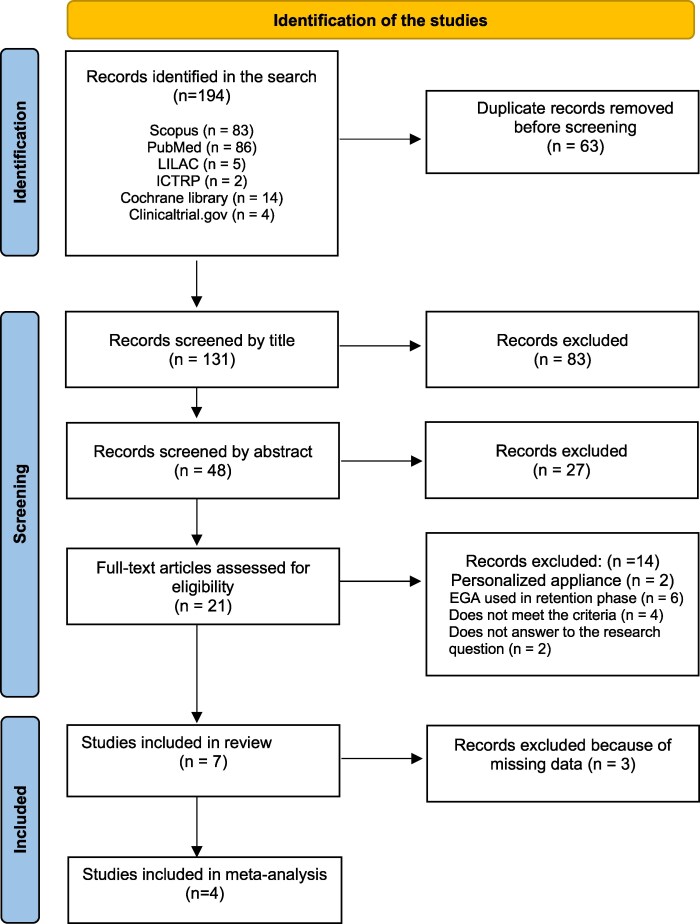
Flow chart describing identification of eligible studies for the systematic review. Edited from Page *et al*. 2020 [10].

After excluding all duplicates and triplicates, two researchers independently screened all the titles and abstracts of the retrieved studies according to the Preferred Reporting Items for Systematic Reviews and Meta-Analyses (PRISMA) guidelines [13]. Any differences between the two reviewers were resolved by consensus-based discussions. The inclusion-exclusion process is shown in Fig. 1. (Excluded articles are shown in Supplementary Table S1.) The full texts of all approved studies were read and judged according to the eligibility criteria. The risk of bias was assessed with ROBINS-I tool (Risk Of Bias In Non-randomized Studies of interventions) in non-randomized studies and with RoB 2 tool in randomized studies [14, 15]. In meta-analyses, the overall certainty of evidence was classified as high, moderate, low, or very low using the Grading of Recommendations Assessment, Development, and Evaluation (GRADE) tool [16].

## Results

**Figure 2:**
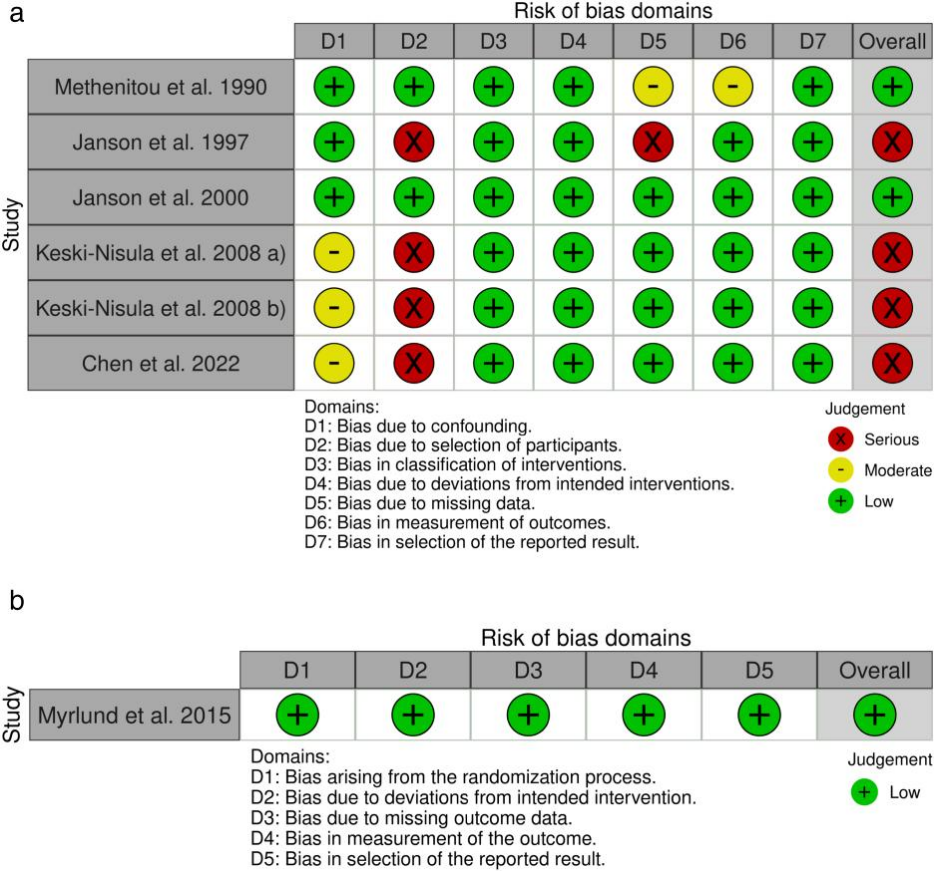
(a) Evaluation of the risk of bias with ROBINS-I tool for prospective and retrospective studies [20]. (b) Evaluation of the risk of bias with RoB 2 tool for the randomized clinical trials [20]. Edited from McGuinness & Higgins 2021 [24].

The electronic literature search produced 131 articles. After a thorough selection process, seven articles were included in the review [17–23]. The effects of the appliances on occlusion and growth pattern were analysed during the early and late mixed dentition stages. The only randomized clinical study had a low risk of bias. The overall risk of bias in the non-randomized studies was found to be either low or serious. The main problems concerned selection of study participants and insufficient information on confounding factors. Four of the studies were limited by selection bias, as only the most cooperative participants were included. Individuals who used the appliance less than instructed were excluded from the analyses. The description of confounding factors was insufficient, and in six of the seven studies, blinding was not mentioned (Fig. 2a and b [24]).

### Study designs

The included seven studies involved a total of 277 patients and 219 controls. Of them, one was retrospective [23], three were prospective treatment studies [17, 19, 21], and one was a randomized controlled trial [22]. Two of the studies were follow-ups analysing cephalometric data regarding patients in clinical studies already included in the review [18, 20]. After contacting the corresponding authors by email, some supplementary values were obtained for the meta-analyses. A summary of the included studies is presented in Table 2.

**Table 2:** Summary of the EGA studies included in the review.

Authors, year	Study design	Case group	Control group	Inclusion criteria (one or more traits)	Type of EGA	Duration (years)	Primary outcome
		*n* Sex Age (years), mean (range or SD)	*n* Sex Age (years), mean (range or SD)				
Methenitou ***et al***., 1990	Prospective	43 26F, 16M 6.2 (3.0–8.7)	50 25M, 25F	Excess OJ Excess OB	Nite-Guide^®^ and Occlus-o-Guide^®^	1.1	EGA preserved a normal OB level. A little or no opening of the face. OJ corrected effectively in primary dentition.
Janson ***et al***. 1997	Prospective	30 13F, 17M 9.0	30 13F, 17M 9.0	Class II, div I (at least end-to-end molar relationship) Class I, excess OB	Occlus-o-Guide^®^	0.8	Overbite and overjet significantly reduced, lower face height reduced, lingual tipping in upper incisors (*P* < 0.001).
Janson ***et al***. 2000	Prospective	30 13F, 17M 9.0	30 13F, 17M 9.0	Class II, div I (at least end-to-end molar relationship) Class I, excess OB	Occlus-o-Guide^®^	2.2	Increase in mandibular growth and lower anterior face height. Improvement in the molar relationships, decrease in OJ and OB.
Keski-Nisula ***et al***., 2008	Prospective	115 53F, 62M 5.1 (SD 0.5)	104 52F, 52M 5.1	Distal step ≥ 1mm Class II canine relationship ≥ 1 mm OB ≥ 3 mm, no tooth contact Deep bite > 3 mm, gingival contact Crowding Anterior crossbite Scissor bite	Occlus-o-Guide^®^	3.3	Occlusal correction, significant increase in mandibular length.
Keski-Nisula ***et al***., 2008	Prospective	167 82F, 86M 5.1 (SD 0.5)	104 52F, 52M 5.1 (SD 0.5)	Distal step ≥ 1 mm Class II canine relationship ≥ 1 mm Crowding OJ ≥ 3 mm, no incisor contact OB ≥ 3 mm, no tooth contact Anterior crossbite Scissor bite	Occlus-o-Guide^®^	3.3	Significant reduction in the frequencies of OJ, OB, open bite, gingival contacts of mandibular incisors, crowding and Class II relationship.
Chen ***et al***., 2022	Retrospective	13 4F, 9M 9.3 (8.1–11.2)	13 4F, 9M 9.9 (8.0–11.7)	Class II, div I OJ > 6 mm CVM ≤ stage III	Education Fonctionnelle (EF)	1.0	Improvement in upper and lower incisors’ inclination.
Myrlund ***et al***., 2015	RCT	24 12F, 12M 7.7 (SD 0.5)	22 10F, 12M 7.7 (SD 0.5)	Class I or Class II with Deep bite (≥ 2/3 overlapping of the incisors) AND/OR OJ ≥ 5 mm AND/OR Moderate anterior crowding with OJ ≥ 4 mm	LM-Activator™	1.0	Improvement in incisal relationships, Class II relationship and crowding.

M, male; F, female; OJ, overjet; OB, overbite; CVM, cerebral vertebral maturation.

### Applied appliances

Four different prefabricated appliances were used: the Occlus-o-Guide^®^ (OG) in five studies, its modification for primary teeth, the Nite-Guide^®^ (NG), followed by the OG in mixed dentition in one study, the Education Fonctionnelle (EF) and the LM-Activator™, in one study each. Instructions for the use of the appliances are presented in Table 3.

**Table 3:** Appliances and instructions for their use in studies included in the review.

Study	Appliance	Instructions
Methenitou ***et al***. (1990)	Occlus-o-Guide^®^ (OG)	Use the appliance during sleep at night. If there are problems with the appliance, wear it during nap time for 1 h. Do not use the appliance during the daytime while awake and do not bite into the appliance.
Janson ***et al***. (1997) Janson ***et al***. (2000)	Occlus-o-Guide^®^ (OG)	Use while sleeping and for 4 h during the day. Daytime use is divided into four 1-h periods. During the first half hour of the period, bite into the appliance firmly for 1 min, then gently for the following minute and so on. The next half hour only bite gently into the appliance, always keeping the lips in contact.
Keski-Nisula ***et al***. (2008) Keski-Nisula ***et al***. (2008)	Occlus-o-Guide^®^ (OG)	Use the appliance during sleeping hours. If there are any problems, use for 1 h during daytime until the nights work out well.
Myrlund ***et al***. (2015)	LM-Activator™	Use every night and for 2 h during the day.
Chen ***et al***. (2022)	Education Fonctionnelle (EF)	Use every night and at least 2 h during the day. Do breathing exercises and lip strength exercises daily.

### Clinical findings

#### Overjet, T1–T2

The overjet reduced from 3.3 to 1.1 mm in 4.5–5.5-year-olds, from 3.0 to 1.4 mm in 6–7.5-year-olds, and from 2.5 to 1.4 mm in 7.5–8.5-year-olds [21]. In the study by Keski-Nisula *et al*. [19], the overjet reduced from 3.1 (SD 1.4) to 1.9 mm (SD 0.7), when treatment was started at the mean age of in 5.1 years (SD 0.5) (*P* < 0.001). In 7-year-olds, Myrlund *et al*. reported a decrease from 4.9 (SD 1.3) to 2.8 mm (SD 1.6) [22]. In 9-year-olds, Janson found a reduction of 2.0 mm (SD 1.3) during the 10 months’ intervention and 2.3 mm (SD 1.5) after 26 months’ intervention [17, 18].

#### Overbite, T1–T2

In the study by Methenitou *et al*. [21], the overbite reduced from 3.4 to 1.4 mm (4.5–5.5-year-olds), from 3.4 to 1.4 mm (6.0–7.5-year-olds), and from 2.6 to 2.0 mm (7.5–8.5-year-olds). All the changes were statistically significant (*P* < 0.01). After the 3.3 years’ treatment period starting at the mean age of 5.1 years, Keski-Nisula *et al*. found a significant reduction in overbite from 3.2 (SD 1.7) to 2.1 mm (SD 0.9) (*P* < 0.001) [19]. In 7.7-year-olds, there was a significant reduction in overbite from 3.4 (SD 1.3) to 2.1 mm (SD 1.3) (*P* < 0.001) [22]. A similar reduction of 1.8 mm (SD 1.3, *P* ≤ 0.01) was reported by Janson *et al*. after 10 months of treatment among 9-year-olds [17]. After 26 months, the mean overbite measured from the cephalograms was 2.0 mm (SD 1.4) less than the clinically measured baseline value (*P* < 0.01) [18].

#### Other changes in occlusion registered clinically or from dental casts

Myrlund *et al*. [22] found that in 9 out of 10 patients, impinging overbite had decreased significantly during the treatment. Keski-Nisula *et al*. found that the number of tooth-to-tooth contacts between the incisors increased from 30 to 165 during the treatment [19].

In the study by Keski-Nisula *et al*. [20], the molar relationship (measured between the distal surfaces of the upper and lower second deciduous molars) decreased significantly from 0.6 (SD 1.7) to −1.3 mm (SD 1.0) (*P* < 0.001). Myrlund *et al*. also found a reduction in the number of patients with Class II molar relationship [22]. Of the original group of 11 patients at T1, only one had Class II molar relationship at T2, and of the eight patients with Class II canine relationships at T1, only one remained Class II [22]. The prevalence of incisor crowding, measured from the dental casts, decreased significantly from 19 to 3 patients in the upper dental arch and from 80 to 2 patients in the lower arch (*P* < 0.001, both jaws) [19].

#### Cephalometric changes

Myrlund *et al*. found significant increases in the angles SNA and SNB [22], upper and lower face heights, facial axis, and the position and inclination of lower incisors, as well as in the inter-incisal angle. In line with Myrlund *et al*. [22], Janson *et al*. [17] found a significant increase in lower anterior facial height (ANS-Me). In their later study, after 26 months of treatment [18], they reported that the vertical components increased significantly (ANS-Me and N-Me), as did the mandibular length (Co-Gn). In line with this, Keski-Nisula *et al*. found a significant increase in the mandibular length (Co-Gn) in the case group [20]. Significant improvements were seen also in the position of lower incisors and in the inter-incisal angle. In the study by Chen *et al*. [23], significant changes were found in the angles SN/upper incisor and upper incisors/nasion–point A (NA) only. Abbreviations of cephalometric variables are shown in Supplementary Table S2.

### Meta-analysis

After a quantitative analysis, four studies were included in the meta-analysis [18, 19, 22, 23]. The reasons for exclusion were missing data, differences in applied references, and reporting styles. The former study by Janson *et al*. [17] was excluded because of partly the same treatment group as in their later study [18].

The pooled estimates for the change were calculated using random-effects model. Hedge’s effect size was calculated for all five analyses, and heterogeneity was evaluated with *I*^2^. The heterogeneity was high for overbite and inter-incisal angle (*I*^2^ > 0.7).

The pooled estimate for overall change in overjet was −1.51 [95% CI −1.74–(−1.28), *P* < 0.01]. The overall change in overbite was −1.67 [95% CI −2.60–(−0.73), *P* < 0.01].

**Figure 3:**
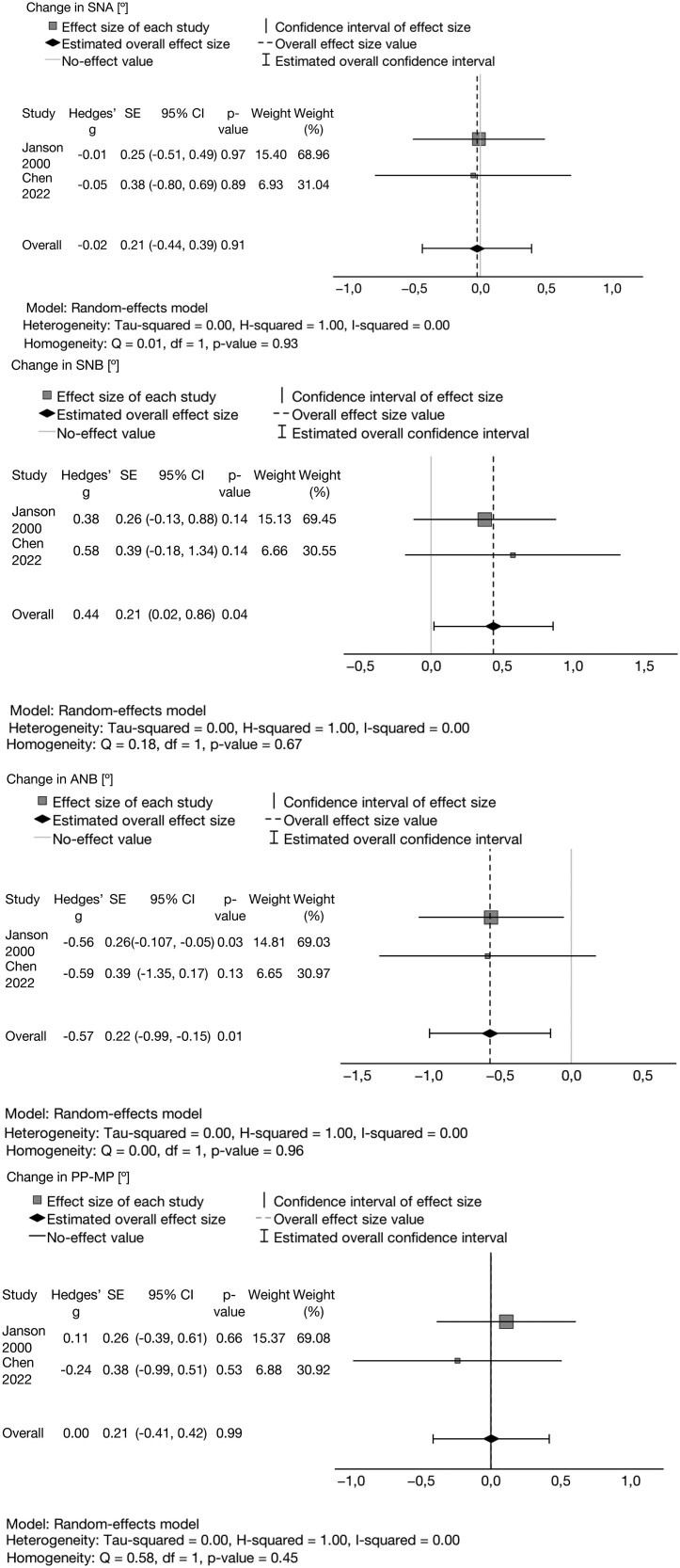
Cephalometric changes in values of SNA, SNB, and ANB in the meta-analysis.

**Figure 4:**
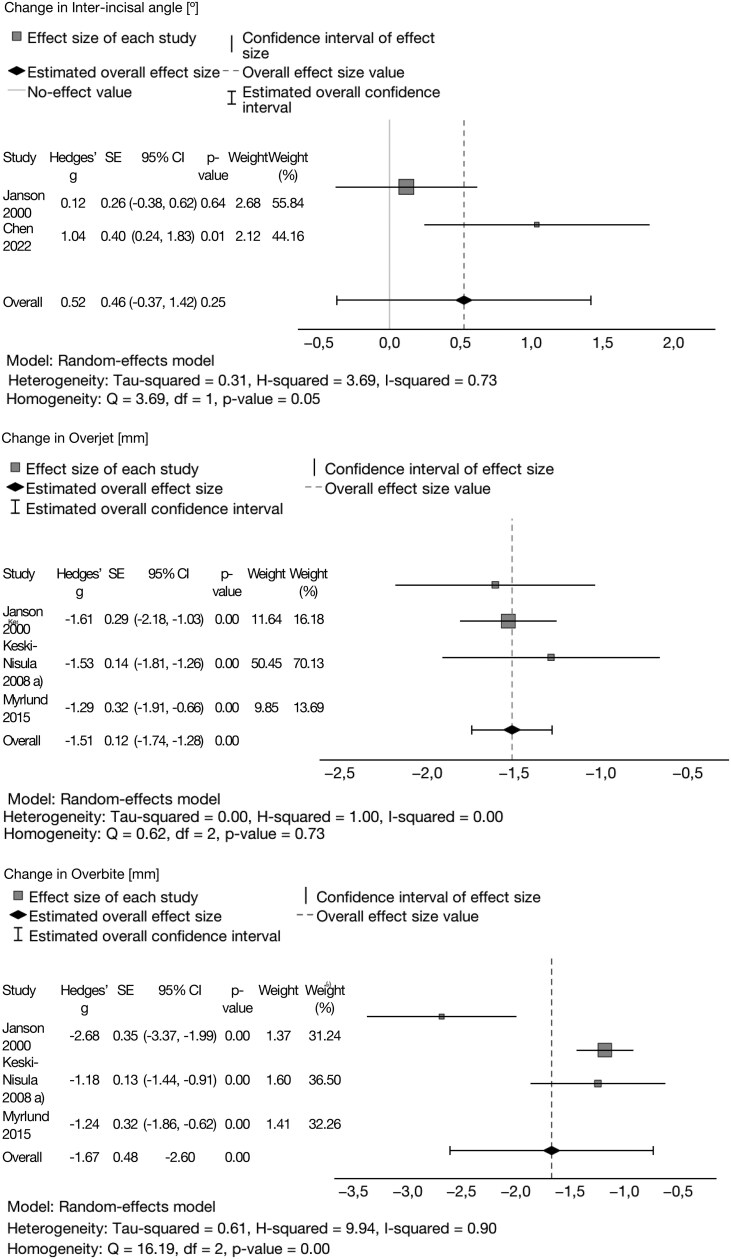
Changes in inter-incisal angle, overjet, and overbite in the meta-analysis.

In the meta-analysis of SNA, the change between T1 and T2 was −0.02 (95% CI −0.44–0.39, *P* = 0.91). Therefore, for SNB, an overall change was estimated at 0.44 (95% CI 0.019–0.86, *P* = 0.04). The included two studies yielded a pooled reduction for the ANB of −0.57 [95% CI −0.99–(−0.15), *P* = 0.01] (Figs 3 and 4). According to the GRADE, the certainty of the evidence was low to very low (Table 4).

**Table 4:** The overall certainty of the evidence in meta-analyses classified using the GRADE tool [16].

Outcome	No. of patients (studies)	Risk of bias	Inconsistency	Indirectness	Imprecision	Publication bias	Difference (95% CI^a^)	Certainty	Comments
Overbite	204 (1 RCT, 2 non-randomized studies)	Serious	Serious	Not serious	Not serious	Undetected	−1.67 (−2.60 to −0.73)	⨁⨁◯◯ Low	Was not downgraded for inconsistency, as heterogeneity was considered clinically irrelevant.
Overjet	204 (1 RCT, 2 non-randomized studies)	Serious	Not serious	Not serious	Not serious	Undetected	−1.51 (−1.74 to −1.28)	⨁⨁◯◯ Low	
SNA	43 (2 non-randomized studies)	Serious	Not serious	Not serious	Serious	Undetected	−0.02 (−0.44 to 0.39)	⨁◯◯◯ Very low	Downgraded due to small sample size.
SNB	43 (2 non-randomized studies)	Serious	Not serious	Not serious	Not serious	Undetected	0.44 (0.02 to 0.86)	⨁◯◯◯ Very low	Downgraded due to small sample size.
ANB	43 (2 non-randomized studies)	Serious	Not serious	Not serious	Not serious	Undetected	−0.57 (−0.99 to −0.15)	⨁◯◯◯ Very low	Downgraded due to small sample size.
PP/MP	43 (2 non-randomized studies)	Serious	Not serious	Not serious	Not serious	Undetected	0.00 (−0.41 to 0.42)	⨁◯◯◯ Very low	Downgraded due to small sample size.
Inter-incisal angle	43 (2 non-randomized studies)	Serious	Serious	Not serious	Serious	Undetected	0.52 (−0.37 to 1.42)	⨁◯◯◯ Very low	Downgraded due to small sample size.

^a^Confidence interval.

## Discussion

EGAs have been used to correct several types of malocclusion, including excess overbite and overjet, Angle Class II division 1 occlusion, anterior crossbite, and anterior crowding [19, 20, 22, 25, 26]. However, scientific evidence of the efficacy of EGAs has been scarce. The aim of this review was to analyse which occlusal traits can be improved with the EGA in the mixed dentition. The second aim was to evaluate skeletal changes.

After a thorough screening for eligibility, seven articles describing the effects of four different EGAs in five different studies were included in the review with both quantitative and qualitative analyses. Reported changes in overbite and overjet confirmed the results of previous reviews [12, 27]: the EGA can improve excessive overbite and overjet mostly by changing the angulation of incisors [12, 22]. Consequently, it also reduces crowding in the mandible [22]. Sagittally, the molar Class II relationships tend to turn towards Class I relationship [20, 22]. Although this correction seems dental rather than skeletal [20], Janson *et al*. and Keski-Nisula *et al*. reported a significant increase in the length of the mandible too [18, 20]. Unfortunately, the available data did not enable a meta-analysis; however, the meta-analysis of the variances in the position of maxilla and mandible (in terms of angles SNA, SNB, and ANB) agrees with the finding.

In addition to the differences in the length of treatment periods, the variability in the results may be explained by several other factors. First is the patients’ age at the beginning of treatment: Methenitou *et al*. reported the best results regarding overbite and overjet in the youngest age group of 4.5–5.0-year-olds [21] and found smaller changes in 7.5–8.5-year-olds. On the other hand, Janson *et al*. found statistically significant improvements in overbite and overjet in 9-year-olds [18], but not in crowding. It may be easier to achieve bigger changes in younger patients in the early mixed dentition stage, because EGAs can literally guide the eruption of permanent teeth [28]. However, it is still possible to obtain favourable results with older patients as well [8].

Like in the retrospective study by Chen *et al*. [23], the studies by Keski-Nisula *et al*. and Janson *et al*. [17–20] included children with the best results after the first 3–10 months of treatment only. Children not using the appliances according to the instructions were excluded.

In two of the included studies, patients were instructed to use the appliance only at night-time, while in others, they were instructed to wear it during daytime, too. Despite this difference, all the studies found significant results, even though the changes among daytime wearers were bigger. However, as shown by Arponen *et al*. [29] and Kutay *et al*. [30], patients do not use their appliance as much as instructed. In the first mentioned study [29], patients who completed treatment used TBA and A-HG appliances on average 45% only of the time prescribed. It is therefore likely that even a rather big increase in the daytime use of EGAs would have a relatively small effect on treatment outcomes. From the clinical point of view, the greatest uncertainty regarding EGA treatments’ success is related to patient compliance. The challenge is same with all removable appliances: the device is effective only when worn. In the studies by Keski-Nisula *et al*. [19] and Nilsson *et al*. [8], one in three patients failed to wear the EGA properly. As stated by Keski-Nisula *et al*. [19], parental support is essential, especially at the beginning of treatment. In comparison to personalized appliances, prefabricated EGAs may not immediately fit perfectly and may, therefore, cause some extra inconvenience. However, in comparison to personalized TBA and A-HG appliances, EGAs are cheaper and have lesser emergency visits [8]. From the clinician’s perspective, microelectronic monitoring could serve as a useful tool for treatment monitoring [29].

In the literature, the suitable length of effective treatment periods has been debated. What treatment period would be long enough to achieve good results, but short enough for young patients to commit to treatment? [5, 31-33] According to Janson *et al*., Myrlund *et al*., and Chen *et al*. [17, 22, 23], the treatment duration should be more than 1 year. A shorter period may not be long enough to observe highly significant changes. In the current review, the total duration of treatment varied between 0.8 and 3.3 years. To ensure that also parental motivation remains sufficiently high, the duration of treatment should not be unnecessarily prolonged.

This systematic review differs from other recent reviews. All study groups had control groups including subjects with similar age and sex distribution. The publications by Papageorgiou *et al*. [10], and Turner et al. [11] had one, and the publication by Huang *et al*. [12] had three common studies with the current review and meta-analysis.

Bearing in mind that the results in treatments with removable appliances are highly dependent on patients’ co-operation, the inclusion of the most compliant patients only is questionable. It may produce the best possible results, instead of giving a realistic view of achievable outcomes on a population basis. From the research point of view, the need for follow-up radiographs poses an ethical problem. In countries like Finland and Norway, it is not considered ethical to take X-rays of controls with no treatment for research purposes only. In this study, missing data and variability in cephalometric measures did not allow for wider meta-analyses. Thus, data enabling cephalometric meta-analyses were unfortunately limited and the certainty of evidence was rated very low. The same problem with scarce cephalometric data was faced in the meta-analysis by Papageorgiou *et al*. [10] too.

To summarize, EGAs comprise a group of prefabricated functional appliances mainly for treatment of Angle Class II division 1 malocclusions. In mixed dentition, they can be used to reduce excessive overjet and overbite. However, more studies are needed to elucidate their skeletal effects.

## Supplementary Material

cjag020_Supplementary_Data

## Data Availability

All data extracted from included studies are shown in Table 2.
