# Supplementary material for: The effect of eruption guidance appliances on occlusal traits: a systematic review and meta-analysis
Source: Eur J Orthod. 2026 May 11;48(3):cjag020. doi: 10.1093/ejo/cjag020 (PMC13159996; doi:10.1093/ejo/cjag020)
Supplement: cjag020_Supplementary_Data [file cjag020_supplementary_data.zip › SUPPLEMENTARY TABLE 1S_07012026.docx]

Supplementary table 1S. Excluded articles with reasons. Abbreviation “EGA” used for Eruption Guidance Appliance.

| AUTHORS | JOURNAL | TITLE | REASON FOR EXCLUSION |
| --- | --- | --- | --- |
| Haydar B, Cier S, Saatçi P. | Am J Orthod Dentofacial Orthop. 1992;102(1):22–8 | Occlusal contact changes after the active phase of orthodontic treatment | Personalised appliance |
| Janson G, Nakamura A, de Freitas MR et al. | Am J Orthod Dentofacial Orthop. 2007;  131(6):729–35 | Apical root resorption comparison between Fränkel and eruption guidance appliances | Does not reply to the study question |
| Park Y, Hartsfield JK, Katona TR et al. | Angle Orthod. 2008; 78(6):1050–56 | Tooth positioner effects on occlusal contacts and treatment outcomes | Personalised appliance |
| Singh S, Singh M, Saini A et al. | J Clin Pediatr Dent. 2010;35(2):233–40 | Timing of myofunctional appliance therapy | Does not reply to the study question |
| Yagci A, Uysal T, Kara S et al. | World J Orthod. 2010;11(2):117–22 | The effects of myofunctional appliance treatment on the perioral and masticatory muscles in Class II, Division 1 patients. | Does not meet the inclusion criteria |
| Stock GJ, McNamara Jr. JA, Baccetti T. | Am J Orthod Dentofacial Orthop. 2011;  140(5):688–95 | Efficacy of 2 finishing protocols in the quality of orthodontic treatment outcome | EGA as a retention appliance |
| Edman Tynelius G, Bondemark L, Lilja-Karlander E. | Orthod Craniofac Res. 2013;16(2): 105–15 | A randomized controlled trial of three orthodontic retention methods in Class I four premolar extraction cases - stability after 2 years in retention | EGA as a retention appliance |
| Tynelius GE. | Swed Dent J Suppl. 2014;236:9–65 | ORTHODONTIC RETENTION. Studies of retention capacity, cost-effectiveness and long-term stability | EGA as a retention appliance |
| Edman Tynelius G, Petrén S, Bondemark L et al. | Eur J Orthod. 2015; 37(4):345–53 | Five-year postretention outcomes of three retention methods - A randomized controlled trial | EGA as a retention appliance |
| Myrlund R, Keski-Nisula K, Kerosuo H. | Angle Orthod. 2019; 89(2):206–13 | Stability of orthodontic treatment outcomes after 1-year treatment with the eruption guidance appliance in the early mixed dentition: A follow-up study | EGA as a retention appliance |
| Keski-Nisula K, Keski-Nisula L, Varrela J. | Eur J Orthod. 2020; 42(2):151-6 | Class II treatment in early mixed dentition with the eruption guidance appliance: effects and long-term stability | EGA as a retention appliance |
| Ciftci V, Uzel A. | Pediatr Dent J. 2021;31(3): 235–41 | Dento-skeletal effects of myofunctional appliance on patients with class II div 1 in mixed dentition stage: A cephalometric study | Does not meet the inclusion criteria |
| Inchingolo AD, Patano A, Coloccia G et al. | [Int J Environ Res Public Health](https://www-scopus-com.ezproxy.utu.fi/sourceid/144989?origin=resultslist) 2022;19(2):988 | The Efficacy of a New AMCOP® Elastodontic Protocol for Orthodontic Interceptive Treatment: A Case Series and Literature Overview | Does not meet the inclusion criteria |
| Lanteri V, Cagetti MG, Ugolin, A et al. | Eur J Paediatr Dent. 2023;24(3): 180–7 | Skeletal and dento-alveolar changes obtained with customised and preformed eruption guidance  appliances after 1-year treatment in early mixed dentition | Does not meet the inclusion criteria |
