# Supplementary material for: The effect of eruption guidance appliances on occlusal traits: a systematic review and meta-analysis
Source: Eur J Orthod. 2026 May 11;48(3):cjag020. doi: 10.1093/ejo/cjag020 (PMC13159996; doi:10.1093/ejo/cjag020)
Supplement: cjag020_Supplementary_Data [file cjag020_supplementary_data.zip › SUPPLEMENTARY TABLE 2S_07012026.docx]

SUPPLEMENTARY TABLE 2S. Abbreviations of cephalometric variables.

*Points*

A Subspinal

ANS Anterior Nasal Spine

B Supramental

Co Condylion

Gn Gnathion

Go Gonion

Me Menton

N Nasion

PNS Posterior Nasal Spine

S Sella

*Lines and planes*

ANS–Me lower face height

Co–Gn mandibular length

MP mandibular plane (Go-Gn)

N–Me total face height

PP palatal plane (ANS–PNS)

SN sella–nasion line (S–N)

*Angular measurements*

ANB relationship between maxilla and mandible in the sagittal plane

Inter-incisal

Angle Angle formed by the long axes of maxillary and mandibular incisors

MP/PP Angle formed by mandibular plane and palatal plane

SNA anteroposterior position of maxilla in relation to anterior part of the cranial base;

SN line to point A

SNB anteroposterior position of mandible in relation to the anterior cranial base;

SN line to point B

SN/

upper incisor angle formed by SN line and axis of upper incisor

upper incisor/

N–point A angle formed by the axis of upper incisor and line nasion (N)–point A
